# Activated Carbon Microsphere from Sodium Lignosulfonate for Cr(VI) Adsorption Evaluation in Wastewater Treatment

**DOI:** 10.3390/polym12010236

**Published:** 2020-01-19

**Authors:** Keyan Yang, Jingchen Xing, Pingping Xu, Jianmin Chang, Qingfa Zhang, Khan Muhammad Usman

**Affiliations:** 1 College of Material Science and Technology, Beijing Forestry University, 35 Qinghua East Road, Haidian District, Beijing 100083, China; ykysdut@163.com (K.Y.); jingchenx612@163.com (J.X.); m18813102213@163.com (P.X.); 2 School of Agricultural and Food Engineering, Shandong University of Technology, 266 Xincun West Road, Zibo 255000, China; zhangqingfacll@126.com; 3 Department of Biological Systems Engineering, Washington State University, Richland, WA 99354, USA; muhammadusman.khan@wsu.edu

**Keywords:** activated carbon microsphere, sodium lignosulfonate, Cr(VI), adsorption

## Abstract

In this study, activated carbon microsphere (SLACM) was prepared from powdered sodium lignosulfonate (SL) and polystyrene by the Mannich reaction and ZnCl_2_ activation, which can be used to remove Cr(VI) from the aqueous solution without adding any binder. The SLACM was characterized and the batch experiments were conducted under different initial pH values, initial concentrations, contact time durations and temperatures to investigate the adsorption performance of Cr(VI) onto SLACM. The results indicated that the SLACM surface area and average pore size were 769.37 m^2^/g and 2.46 nm (the mesoporous material), respectively. It was found that the reduced initial pH value, the increased temperature and initial Cr(VI) concentration were beneficial to Cr(VI) adsorption. The maximum adsorption capacity of Cr(VI) on SLACM was 227.7 mg/g at an initial pH value of 2 and the temperature of 40 °C. The adsorption of SLACM for Cr(VI) mainly occurred during the initial stages of the adsorption process. The adsorption kinetic and isotherm experimental data were thoroughly described by Elovich and Langmuir models, respectively. SL could be considered as a potential raw material for the production of activated carbon, which had a considerable potential for the Cr(VI) removal from wastewater.

## 1. Introduction

Water pollution has become a global issue because of its increasing impact on human and animal health. The presence of heavy metals in industrial water is not only causing severe damage to human and animal health, but it is also damaging aquatic life [1,2,3]. The effluents from a variety of industrial processes such as metallurgy, petroleum refining, batteries, and electroplating are responsible for introducing these heavy metals into aquatic environments [4,5]. These heavy metals include chromium (Cr), cadmium (Cd), lead (Pb), mercury (Hg), and other heavy metal ions and compounds [6,7,8]. These heavy metals are insistent and non-degradable in nature, but they are soluble in the aquatic environment; therefore, they can be easily absorbed into living cells [9]. At the same time, contamination of water by heavy metals is also considered one of the main reasons for the non-availability of potable water in developing and underdeveloped countries [10]. Among all of the heavy metals, Cr is one of the main 16 toxic metals considered detrimental for human health [11]. Cr(III) is a human micronutrient, while Cr(VI) is extremely toxic and is a strong oxidizing agent [12]. The presence of Cr(VI) in water can cause severe diseases, such as kidney circulation, dermatitis and lung cancer [13,14]. Therefore, removal of Cr(VI) from aquatic environments is indispensable for public health, as well as for the protection of the environment and aquatic life. Moreover, strict environmental mechanisms and the enactment of legal standards is necessary to avoid excessive discharge of Cr(VI) into potable water sources [15]. The Environmental Protection Agency (EPA) has recommended that the maximum concentration of Cr(VI) in drinking water not exceed more than 0.05 mg/L [12].

Many techniques, such as adsorption, membrane filtration, electrodialysis ion exchange, reduction, reverse osmosis and biological removal have been developed to remove Cr(VI) from aquatic environments [13,16,17]. Among them, adsorption is one of the most effective methods and is suitable for use in developing countries by using adsorbents [18]. Adsorption is a green and low-cost wastewater treatment technique, especially useful for heavy metal ions and hydrophilic compounds such as chromium, lead, ammonium ions and antibiotics [10,19,20,21]. The adsorbent generally has an abundant pore structure, while simultaneously having chemical functional groups on the surface, and it can be easily modified through increased surface charge [18]. In terms of removing chromium ions from wastewater, the adsorbent used by the adsorption method has a lower cost than the ionic exchange resins used by membrane filtration and the membranes used by electrodialysis ion exchange; furthermore, adsorption is easier to perform than reduction and reverse osmosis, and it has a higher processing efficiency and more extensive application fields than biological removal. In general, compared to other methods, activated carbon absorption has been proved to be a preferable technique for the removal of Cr(VI) from wastewater due to its higher efficiency, lower operating cost, higher adsorption capacity, easier operation, and non-hazardous technique. Thus, the activated carbon adsorption process has been developed and applied extensively [21,22].

Currently, the raw material to produce activated carbon comes from conventional fossil fuels such as coal and petroleum. With the development of renewable energy sources, the exploration of novel and effective adsorbents based on renewable sources has also received more attention than ever before due to the growing concerns about environment and increasing cost of fossil fuels [23]. Keeping in mind the environmental and industrialization concerns, it is necessary to develop activated carbons from low-cost and abundant precursors. During the last few years, many researchers have used adsorbents made of different types of renewable biomasses such as tobacco stems [24], longan seed [4], rice straw [25], juniperus procera sawdust, avocado kernel seeds and papaya peels [26], activated carbon derived from leucaena leucocephala [27], and sterculia guttata shell [28], and the results of these studies indicates that these adsorbents are helpful in achieving sufficient removal of Cr(VI) from wastewater. However, the cost of these adsorbents was found to be higher, due to which these adsorbents are not being used at commercial scale.

Therefore, a novel, cost-effective and efficient activated carbon is highly needed for proper and cost-effective treatment of wastewater at larger scales. The SL is an inevitable by-product of the paper and pulp industry. Currently, most of the SL generated by the paper and pulp industry is burnt or dumped into open lands and drains, which is not only causing an increase environmental pollution, but also wastes a valuable resource. It is necessary to achieve high-value utilization of SL, rather than wasting it. In addition to high carbon content, SL contains oxygenic functional groups such as carboxyl and phenol hydroxyl [29]. Moreover, some researchers have also found that SL and its derivatives have remarkable electrochemical performance and the potential to adsorb certain heavy metals, including toluene, and some other pollutants [30,31]. However, the problem associated with SL as an adsorbent is its high solubility in water, due to which it is difficult to remove powder SL from aqueous solution after the adsorption process, which can cause secondary pollution. That is a common reason for limiting powder SL application as adsorbing material. To solve the separation problem of powder SL after adsorption from solution, magnetic lignosulfonate was fabricated based on magnetic separation technique [32]. However, beyond that, changing the macroscopic character of SL from powder to granular to prepare granular activated carbon is a valuable research direction for solving the separation problem, as well as for improving the adsorption properties of SL. The activated carbon prepared by conventional methods from SL is also powdered. However, the powdered activated carbon is very difficult to apply in wastewater treatment, because it is dispersed in the water and very hard to separate after adsorption. If the powdered activated carbon is to be applied commercially, then it must be prepared into granular activated carbon by means of a binder. However, the application of binder is costly and environmentally unfriendly. Therefore, a new preparation method for activated carbon microspheres from SL needs to be developed. Because the SL has good reaction activity, it could be used to react with basis materials such as polystyrene to generate large particles of adsorption material with easy recovery and separation, in order to realize high-value industrial utilization of SL as an adsorption material. The SL also has lower cost than many other fossil fuels as a kind of renewable biomass resource with vast reserves. Therefore, the SL that is considered to be waste in the paper and pulp industry and is generally dumped into the open environment, causing an increase in environmental pollution, can be used as a renewable precursor to substitute traditional fossil fuels for the production of activated carbon, which can then be used as a low-cost adsorbent for the removal of heavy metals from polluted aquatic environments.

Keeping in mind the acute need for low-cost and efficient renewable adsorbents in the wastewater treatment sector and the promising behavior of adsorbents prepared from biomass, in this study the SLACM was developed, aiming to remove Cr(VI) from wastewater. The preparation of SLACM was done in two steps, i.e., the preparation of activated carbon precursor from powdered SL and polystyrene by Mannich reaction without the addition of any binder, and the ZnCl_2_ activation of the precursor. The effects of the adsorption process on the adsorption capacity of SLACM for Cr(VI) were investigated according to batch experiments carried out under different initial pH, initial concentration, contact time, and temperature. The prepared SLACM was characterized with respect to its pore volume, surface area, and sorption efficiency, and the transformation of structure and properties before and after Cr(VI) adsorption was compared. On the basis of the ability to remove Cr(VI) in the solution experiments, adsorption kinetics, and isotherm fitting, the potential of using SLACM for the purification of wastewater was evaluated.

## 2. Materials and Methods

### 2.1. Materials

The SL was purchased from Shanghai Macklin Biochemical Co., Ltd., Shanghai, China. The SL was composed of 41.63% carbon, 28.32% oxygen, and 24.39% sodium, as well as small amounts of silicon. The chloromethylated polystyrene was used as the base material in the preparation. chloromethylstyrene-divinylbenzene-styrene copolymer (CMPS) was purchased from Tianjin Xingnan macromolecule technology Co., Ltd., Tianjin, China. 1,3-diaminopropane (C_3_H_10_N_2_, MW 74.13) was purchased from Shanghai Macklin Biochemical Co., Ltd. Tetrahydrofuran (C_4_H_8_O) was purchased from Beijing Chemical Co., Ltd. Beijing, China. Formaldehyde (HCHO) was purchased from Xilong Scientific Co., Ltd., Guangdong, China. ZnCl_2_ was purchased from Shanghai Macklin Biochemical Co., Ltd., Shanghai, China. Methyl orange was purchased from shanghai D&B biological science and technology Co., Ltd., Shanghai, China. Deionized water was used for all the experiments. All chemicals and materials were of analytical grade and were used without further purification.

### 2.2. Preparation of SLACM

**Figure 1:**
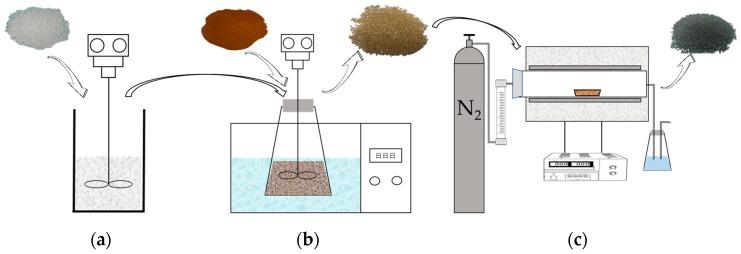
The preparation process diagram of SLACM: (**a**) Amination of CMPS; (**b**) Mannich reaction of SL and ACMPS; (**c**) Activation processes.

**Figure 2:**
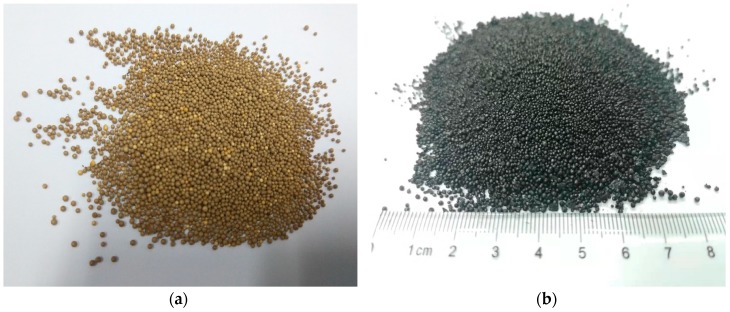
(**a**) The ARM sample picture; (**b**) The SLACM sample picture.

The preparation of SLACM was done in two main steps, i.e., preparation of activated carbon precursor from powdered SL and polystyrene by Mannich reaction and the ZnCl_2_ activation of the precursor. The preparation process diagram of SLACM was shown in Figure 1. The substrate material CMPS need to be amination pretreated before reaction. The CMPS was first swollen 2 h with tetrahydrofuran (3 mL/g) and then amination treated for 12 h in the 50 °C with 1,3-diaminopropane (5 mL/g). After that, the SL and amino CMPS ware generate large particles of adsorbent resin microsphere (ARM) by Mannich reaction in solution catalyzed by formaldehyde at a mass ratio of 1:1. The Mannich reaction of SL and amino CMPS continuously proceed for 12 h in the 90 °C water bath. The ARM samples are shown in Figure 2a. The ARM was oxidized for 30 min at 180 °C before the impregnation process to obtain pre-oxidized adsorbent resin microsphere (PARM) as an activated carbon precursor. A suitable amount of PARM was added to the activator aqueous solution (50 wt.%), and the solution was stirred continuously at room temperature to prepare a uniform solution. ZnCl_2_ was used as an activating agent during the impregnation process with an impregnation ratio of 1:1 of activated carbon precursors to activator respectively. After 12 h of the impregnation process, the samples were dried at 105 °C for 6 h in an oven. The impregnated activated carbon precursors were put into a porcelain boat in a horizontal tube furnace and heated with nitrogen (99.99%) protected. During the activation process, the temperature was initially increased from room temperature to the activation temperature of 600 °C by using a heating rate of 10 °C/min, and then the temperature was kept constant at 600 °C for 2 h. After this, the samples were cooled to room temperature under nitrogen atmosphere. Finally, the samples were washed 2–3 times with rare hydrochloric acid (0.5 mol/L) and deionized water in sequence until the pH of filtrate became neutral and then dried at 105 °C for 12 h to obtain dry SLACM. The SLACM samples are shown in Figure 2b.

### 2.3. Characterizations of SLACM

The surface morphology of SLACM samples was observed by scanning electron microscopy (SEM, Hitachi S-4800, Hitachi, Japan) at an accelerating voltage 5.00 kV and the element chemistry configuration was analyzed by SEM with energy dispersive spectrometry (EDS) system. The microstructures of the SLACM samples was characterized by transmission electron microscope (TEM, FEI TF30, Hillsboro, OR, USA). A physisorption analyzer (Quantachrome autosorb-IQ, Boynton Beach, FL, USA) was used to determine the specific surface area, pore volume and other pore characteristics of SLACM samples. The SLACM samples pulverized to carbon powder were treated by vacuum degassing 3 h at 200 °C at first, and then N_2_ adsorption and desorption isotherms were measured at 77 K. The specific surface area and pore volume of SLACM samples were analyzed by the BET method according to the N_2_ adsorption and desorption isotherms. The BET equation was applied to N_2_ adsorption data from 0.06 < P/P_0_ < 0.25 to determine surface area and total pore volume was measured at P/P_0_ = 0.985. The surface functional groups of SLACM were characterized before and after adsorption by using a Fourier transform infrared spectrometer (FT-IR, Thermo Scientific Nicolet iS10, San Jose, CA, USA) in the range from 4000 to 400 cm^−1^ at 4 cm^−1^ resolutions with 64 scans. The crystalline structure of SLACM specimen was investigated by X-ray diffraction (XRD, Bruker D8 ADVANCE diffractometer) with Cu Kα radiation (λ = 0.15417 nm) in the range of 2*θ* = 10–90 at a step rate of 0.02. The mass change of activated carbon precursors was measured by the thermogravimetric analysis (TG) (STA449C, Selb, Germany) under an N_2_ atmosphere from 30 to 800 °C at the rate of 10 °C/min.

### 2.4. Adsorption Experiment

All batch experiments were carried out in 250 mL Erlenmeyer flasks with 100 mg SLACM and 50 mL Cr(VI) solution in order to study the adsorption capacity of adsorbent on Cr(VI). Several experimental parameters which may affect the absorption efficiency of SLACM were studied, including initial pH, contact time, temperature and initial concentration of Cr(VI). When the SLACM and Cr(VI) solution was taken in the flasks, HCl solution (0.1 mol/L) and NaOH solution (0.1 mol/L) was used to adjusted the initial pH of the solution to setting value. The Erlenmeyer flasks were then placed on a 150-rpm shaking table to determine the adsorption capacity of SLACM according to setting different contact time and temperature. After the designated time of adsorption, the mixture was separated using a 0.45 μm filter. The filtrate absorbance was measured by UV-Vis spectrophotometer, and Cr(VI) concentration was calculated according to the diphenyl carbohydrazide spectrophotometric method (Chinese National Standards GB/T 7467-87). All the adsorption experiments were repeated 3 times and the average values were used as final results.

The adsorption capacity $q_{t}$ and $q_{e}$ of Cr(VI) onto SLACM were calculated as follows:

(1) $$q_{t}=\frac{\left(C_{i}-C_{t}\right)V}{M},$$

(2) $$q_{e}=\frac{\left(C_{i}-C_{e}\right)V}{M},$$

where $q_{t}$ (mg/g) is the adsorption capacity at time t, $q_{e}$ (mg/g) is the adsorption capacity at equilibrium, $C_{i}$ (mg/L) is the initial concentration of Cr(VI), $C_{t}$ (mg/L) is the concentration of Cr(VI) at time t, $C_{e}$ (mg/L) is the concentration of Cr(VI) at equilibrium, V (L) is the volume of suspension of Cr(VI), M (g) is the dry weight of the SLACM.

#### 2.4.1. Effects of Initial pH on Adsorption

The effects of initial pH on the Cr(VI) adsorption were studied by adding SLACM to flasks with 150 mg/L Cr(VI) solution under the similar experimental conditions. The pH value of the solution was then adjusted to 1.0, 2.0, 3.0, 4.0, 5.0, 6.0, 7.0, 8.0 and 9.0 by using HCl solution (0.1 mol/L) and NaOH solution (0.1 mol/L). All the samples were shaken in a shaking table for 12 h (30 °C, 150 rpm).

#### 2.4.2. Adsorption Kinetics

The adsorption kinetics of Cr(VI) on the SLACM was studied by using a contact times ranging from 2 min to 24 h with initial Cr(VI) concentration of 150 mg/L and pH value 2. The agitation speed and the contact temperature of the shaking table were kept constant at 150 rpm and 30 °C, respectively.

#### 2.4.3. Adsorption Isotherm

Adsorption isotherms are usually used to describe the distribution of metal ions between the liquid phase and the solid phase [33]. Adsorption isotherms were performed for 20, 30 and 40 °C by using initial Cr(VI) concentrations, pH, contact time, and a shaking speed of 100 to 600 mg/L, 2, 12 hand 150 rpm, respectively.

## 3. Results and Discussion

### 3.1. Characterization

The pore structure of activated carbon is an important factor influencing adsorption capacity. The specific surface area and pore volume of SLACM sample was evaluated by the BET method according to Nitrogen adsorption isotherms measured at 77 K. The porous structure parameters of SLACM are shown in Table 1. It is clear from the table that the BET surface area of SLACM is 769.37 m^2^/g and the total pore volume is 0.47 cm^3^/g. The chromium ions could be trapped in the pores of SLACM due to the radiuses of chromium ions were less than the pore size of the adsorbent [33]. The well-developed BET surface area and the total pore volume mean that the absorption performance of SLACM is good and it can be used as a substitute to conventional adsorbents. The average pore size of SLACM was found to be 2.46 nm, and thus it belongs to the mesoporous materials.

**Figure 3:**
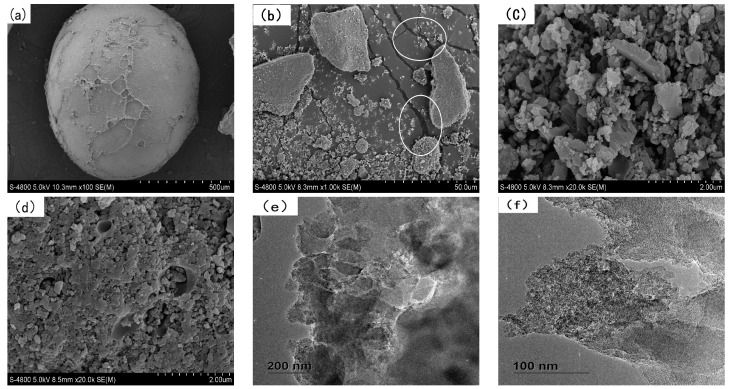
(**a**,**b**) SEM images of SLACM surface before Cr(VI) adsorption of 100 and 1 k; (**c**) SEM image of SLACM fracture surface before Cr(VI) adsorption of 20 k; (**d**) SEM image of SLACM fracture surface after Cr(VI) adsorption of 20 k; (**e**,**f**) TEM images of SLACM before and after Cr(VI) adsorption.

**Figure 4:**
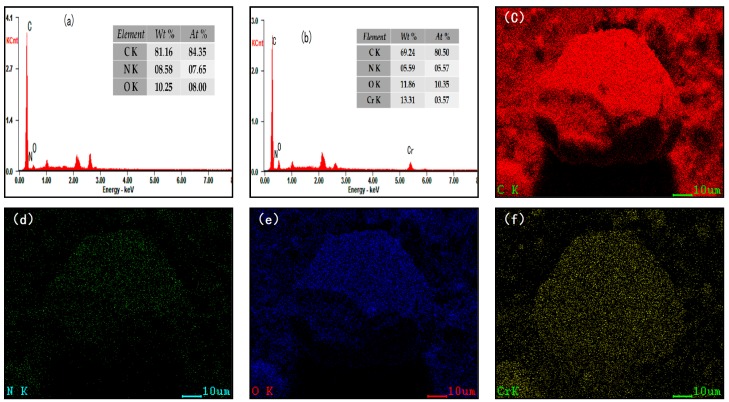
(**a**) EDS spectrum of SLACM before Cr(VI) adsorption; (**b**) EDS spectrum of SLACM after Cr(VI) adsorption; (**c–f**) EDS elemental mapping patterns of C, N, O and Cr after Cr(VI) adsorption.

The surface microstructure and fracture micro appearance of SLACM was characterized using SEM and TEM, the surface microstructure of SLACM, fracture micro appearance of crushed SLACM before and after adsorption, the TEM images of SLACM before and after adsorption are presented in Figure 3. As shown in Figure 3a, the SLACM was a regular sphere and the diameter of most particles was around 0.5–1.5 mm. Due to its larger size, the activated carbon can be separated out more easily from solution after adsorbing than the powdery SL. Thus, the recycling properties as well as convenience of use of the activated carbon were improved significantly. There were a lot of different size clearances and cracks present on the SLACM surface, as shown in Figure 3b, and these were the main channels through which the adsorbate could enter inside [10]. The well-developed pore structure should be responsible for the adsorption of Cr(VI) onto SLACM. It was also found by the fracture micro appearance of inner side of SLACM that the abundant pores and cavity structure confirmed the high specific surface areas of adsorbent, this could also be supported by the porous structure parameters of SLACM. The TEM image confirms that the SLACM before adsorption is hollow, and this observation is in line with SEM fracture surface image. As can be seen from Figure 3d,f, the pores of SLACM were blocked, and obvious attachment appeared on the sample surface after Cr(VI) adsorption. The EDS spectrum of the SLACM before and after Cr(VI) adsorption and EDS elemental mapping patterns of C, N, O and Cr of SLACM after Cr(VI) adsorption were shown in Figure 4. It was observed that carbon, nitrogen and oxygen were the major elements in SLACM and that carbon, nitrogen, oxygen and chromium were the major elements on SLACM after Cr(VI) adsorption. The elements of nitrogen and oxygen have a greater promoting effect in the adsorption which could enhance the adsorption efficiency of SLACM for Cr(VI) [34]. The SLACM after adsorption showed distinct peaks of chromium present on the surface and the EDS mapping reveals that the chromium was evenly distributed over SLACM surface. Therefore, EDS analysis confirmed the adsorption of Cr(VI) onto SLACM.

**Figure 5:**
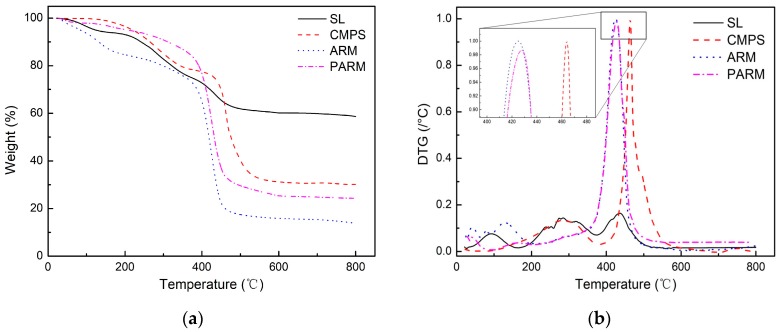
(**a**) The TG curves of SL, CMPS, ARM and PARM; (**b**) The DTG curves of SL, CMPS, ARM and PARM.

The thermal behavior of the CMPS, ARM and PARM was analyzed under nitrogen atmosphere. The TG and derivative thermogravimetric (DTG) curves of SL, CMPS, ARM and PARM are illustrated in Figure 5. The SL has a high residual mass rate after heating and a low peak value of DTG than others. A high residual mass rate is positive for preparing of SLACM. Compared with CMPS, the maximum weight loss temperature (*T*_max_) of AR was reduced more significantly. This is due to the destruction of part of long chain structure or aromatic substitution during the graft polymerization of SL and CMPS, the protection of lignin was break down [35]. For these reasons, the residual mass rate of ARM is much less than that of SL and CMPS. The residual mass rate of PARM has a larger increase compared to ARM, probably because of partial structural carbonization or pyrolysis in ARM during the oxidation process [36]. Keeping in mind the larger increase in the residual mass rate of PARM, it can be concluded that a higher yield SLACM can be prepared by using PARM as raw material compare to ARM. It is also clear from Figure 5 that the residual mass rate of all the samples did not decrease when the temperature was higher than 600 °C, which suggests that the activation temperature should not be increased to higher than 600 °C during the preparation of SLACM. Thus, the activation temperature for SLACM was found to be lower than activated carbon production from other studies in the literature [33,37,38,39], which can save a large amount of energy during production process.

**Figure 6:**
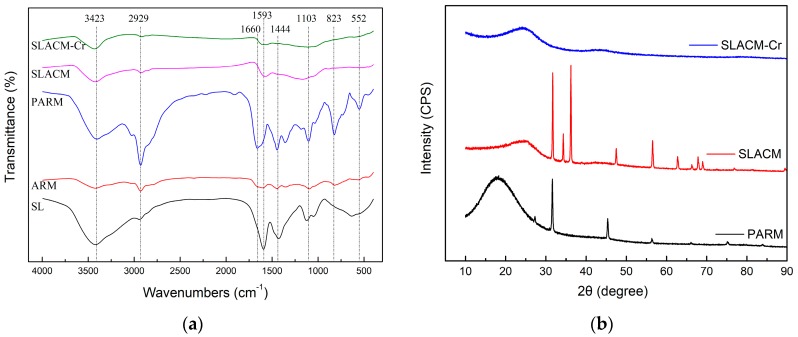
(**a**) The FT-IR spectra of samples; (**b**) The XRD curves of samples.

The structural variation of the SLACM samples and other materials were analyzed by FT-IR and the recorded spectra are shown in Figure 6a. The wide peak at 3423 cm^−1^ corresponding to stretching of aliphatic and phenolic −OH groups was observed in all of the samples, especially SL. The peaks at 1593 and 1444 cm^−1^ were typical of aromatic C–C stretching and C–H bending in SL. The peak intensity of SL at 3423 and 1593 cm^−1^ was much stronger than ARM, which meant aromatic phenolic -OH groups in SL were combined with aminated CMPS after Mannich reaction. After the pre-oxidation, new peaks appeared in PARM. The peak around 2929 cm^−1^ was attributed to the stretching vibration of saturated hydrocarbon –CH_3_, –CH_2_– and the methyl or methylene in aliphatic groups. The peak at 1660 cm^−1^ was attributed to the stretching vibration of amide –CO–N [31]. This may be because the –NH oxidizes in AR and reaction produced -CO-N groups. The peak at 1103 cm^−1^ was attributed to the antisymmetric stretching vibration of S–O in SO^3−^ groups, and the SO^3−^ group was from SL [32]. This indicates that SL was successfully grafted onto aminated CMPS. After activation, the peak amount of SLACM was significantly less than PARM and the peak intensity was obviously weaker than PARM. This was because some chemical bonds of PARM were broken down at higher temperature, such as alken-CH_3_, aliphatic ether –C–O–, and the benzene ring replacing op (CH), and forming graphitized carbon materials [31]. Some small weak peaks appeared at 3423 and 1593 cm^−1^ on the SLACM curve. These results show that SLACM contains a variety of functional groups including phenols, alcohols, alkenes, amines, etc. The main functional groups are in good agreement with biomass-activated carbon from others [31,40]. The oxygen-containing functional groups of SLACM determine the surface acidity-basicity and the adsorption performances. After adsorption of Cr(VI), these peaks of SLACM-Cr were not obvious, indicating an interaction between the Cr (VI) and SLACM.

The XRD spectra representing the crystalline structures of PARM, SLACM, SLACM-Cr (after adsorption of Cr(VI)) are shown in Figure 6b. The results show that PARM has an amorphous wide peak at 18.28°. The wide peak of the SLACM decreased and moved to the right compared to PARM. This illustrates that the distance of crystal face of SLACM decreased and the graphitization degree increased after activation at high temperature [33,41]. The diffraction peaks of SLACM occurred at 31.72°, 34.35°, 36.2°, 56.54°. This is because the crystal structure of samples changed at higher temperature, main performance for small particle graphite structure and undefined structure. The diffraction peaks of SLACM-Cr disappeared after the Cr(VI) adsorption. This was due to fact that the Cr(VI) was adsorbed successfully by SLACM as a physical or chemical form and the adsorbate Cr(VI) damaged the crystal structures.

### 3.2. Effect of Initial pH on Adsorption

**Figure 7:**
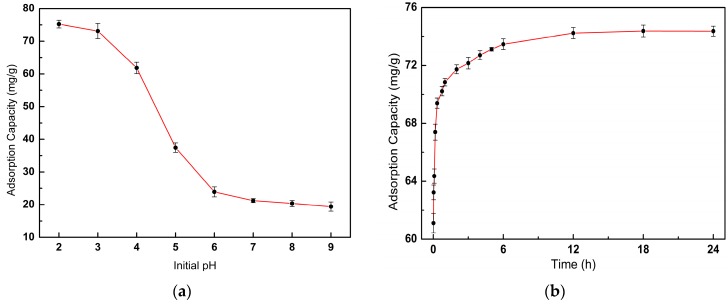
(**a**) Effect of initial pH on adsorption capacity of SLACM for Cr(VI); (**b**) Effect of time on adsorption capacity of SLACM for Cr(VI).

The initial pH of the solution is an important parameter that influences the adsorption capacity of the adsorbent for metal ions [42]. The effect of initial pH on the adsorption capacity of SLACM for Cr(VI) was tested, and the results are shown in Figure 7a. It is clear from the results that adsorption capacity of SLACM for Cr(VI) is strongly dependent on the initial pH of the solution. Moreover, the adsorption capacity of SLACM continuously decreases as the pH increases from 2.0 to 9.0. This result is comparable with other consequences reported in the literature [43]. Cr(VI) exist in the form of oxyanions in solution, and the existence form of Cr(VI) ion depends mainly on the solution pH value. The predominant Cr(VI) existence forms are dichromate (Cr_2_O_7_^2−^) and hydrogen chromate (HcrO^4−^) when the pH of the aqueous solutions was in range of 2.0–6.0. When the pH exceeded 7.0, chromate (CrO_4_^2−^) was the main Cr(VI) species [33,37]. The initial pH of the solution simultaneously affects the adsorbent surface charge and degree of ionization [44]. At lower pH, the functional groups on the surface of the SLACM were protonated by plentiful hydrogen ions, and there was a strong electrostatic interaction between the negatively charged Cr(VI) and the positively charged SLACM. The strong electrostatic interaction was very favorable for adsorbing dichromate and hydrogen chromate. Cr(VI) could be reduced to Cr(III) with the SLACM surface charge provided by the oxygen-containing functional groups, such as -C-O- and -OH. The Cr(VI) was adsorbed by complexation or reduced may be described by the following reactions (Equations (3)–(6)) [13,33]. The adsorbed Cr(VI) was reduced to Cr(III) by the electron-donor groups of the porous carbon from corn straw was also proved by Ma et al. [33]. Therefore, the highest adsorption capacity of SLACM for Cr(VI) was the 75.25 mg/g when the initial pH of solution was 2.0. As the initial pH of the solution increased, the protonation degree of the SLACM surface functional groups decreased and gradually converted to negatively charged. There exists competitive adsorption between OH- and chromate, which interferes with the binding site on the adsorbents; the OH- content in solution increased, and the competitive adsorption was found to be stronger at higher pH values. Electrostatic repulsion and competitive adsorption caused a decrease in the adsorption capacity of SLACM for Cr(VI); therefore, the adsorption capacity of SLACM for Cr(VI) dropped to 19.42 mg/g when the pH was raised to 9.0. Moreover, Cr(VI) and the –COOH group of SLACM may have an ion exchange reaction (Equation (7)). These results indicate that the Cr(VI) removal behavior was affected by the initial pH of solution, and pH 2.0 was applied to the following adsorption experiments to obtain the optimal adsorption performance in this study.

(3) $${\mathrm{RO}}^{-}+{\mathrm{Cr}}^{6+}\to {\mathrm{RO}}^{-}\ldots {\mathrm{Cr}}^{6+}$$

(4) $$3\mathrm{ROH}+{\mathrm{Cr}}_{2}O_{7}^{2-}+4H^{+}\to 3\mathrm{RO}+{\mathrm{HCrO}}_{4}^{-}+{\mathrm{Cr}}^{3+}+3H_{2}O$$

(5) $$3\mathrm{ROH}+{\mathrm{HCrO}}_{4}^{-}+4H^{+}\to 3\mathrm{RO}+{\mathrm{Cr}}^{3+}+4H_{2}O$$

(6) $$3C-O-+3{\mathrm{HCrO}}_{4}^{-}+5H^{+}\to 3C-O+3R\ldots {\mathrm{Cr}}^{3+}+4H_{2}O$$

(7) $$3R-\mathrm{COOH}+{\mathrm{Cr}}^{6+}\to R-{\mathrm{COO}}^{-}\ldots {\mathrm{Cr}}^{6+}+H^{+}$$

### 3.3. Adsorption Kinetics

To evaluate the kinetic behavior of adsorption process of SLACM for Cr(VI), the adsorption time of SLACM in solution was set as 2 min to 24 h. The adsorption velocity of SLACM for Cr(VI) was different at different time points during the adsorption process, but eventually it all balanced out. The adsorption kinetics were determined in order to study the relationship between the adsorption capacity and adsorption time during the adsorption process, and were significant in explaining the adsorption process in view of the order of the rate constants. The relationship of adsorption capacity of SLACM for Cr(VI) and adsorption time are shown in Figure 7b. As can be seen, the adsorption capacity of SLACM for Cr(VI) increased rapidly in the first hour and was 70.84 mg/g after 1 h. The increase in adsorption capacity slowed down between 1 and 6 h, and then the adsorption capacity gradually reached equilibrium. The equilibrium adsorption capacity was 74.38 mg/g. The pseudo first-order (PFO), pseudo second-order (PSO) and Elovich models were applied to the experimental data to evaluate the adsorption kinetics, in order to understand the controlling mechanisms of the Cr(VI) sorption on SLACM [7,27]. These models are given as the following equations, respectively.

(8) $$\mathrm{PFO}:\;q_{t}=q_{e}\left(1-e^{-k_{1}t}\right),$$

(9) $$\mathrm{PSO}:\;q_{t}=\frac{q_{e}^{2}k_{2}t}{1+q_{e}k_{2}t},$$

(10) $$\mathrm{Elovich}:\;q_{t}=\frac{1}{\beta }\ln\left(1+\alpha \beta t\right),$$

where $q_{t}$ (mg/g) is the adsorption capacity at t time, $q_{e}$ (mg/g) is the adsorption capacity at t time at equilibrium, t (h) is adsorption capacity time, $k_{1}$ (min^−1^) is the PFO equilibrium rate constant, $k_{2}$ (g/mg·min) is the PSO equilibrium rate constant, α and β are Elovich constants. The adsorption kinetic constants and dynamic fitting parameters were summarized in Table 2. The fitted results in Table 2 show that the Elovich model is in better agreement with the experimental results when explaining adsorption rate than the PFO and PSO models, indicating the better suitability of Elovich model for describing the adsorption kinetics of Cr(VI) onto SLACM. Therefore, the adsorption type of Cr(VI) onto SLACM was determined to be predominantly chemisorption, which was consistent with heavy metal ion adsorption results using different solid adsorbents, as described in other literature studies [7]. The adsorption of SLACM includes mass transfer of Cr(VI) to the external and internal surface of adsorbent, particle diffusion, active site adsorption of adsorbent and other multi-stages adsorption process [40]. The adsorption process can be divided into several stages. The Cr(VI) in solution firstly diffused to the SLACM surface then it entered inside the adsorbent through pores. The adsorbate was adsorbed by pore structures and the active sites on external and internal surface of SLACM during these processes. The available active sites on the surface was sufficient and the pore structure was not completely filled during the initial stages of adsorption process, the mass transfer resistance of Cr(VI) was small. This was due to the presence of a greater driving force provided by the higher initial concentration of Cr(VI), which increased the probability of collisions between Cr (VI) and the active sites. Therefore, the adsorption process has a high adsorption rate at the beginning. The active sites on the surface were inhibited and the pore structure was filled gradually through the process of adsorption. Therefore, the interactions between Cr(VI) and active sites decreased and the mass transfer resistance of adsorbate increased. As shown in the curve in Figure 7b, the adsorption rate decreased and the adsorption capacity of SLACM for Cr(VI) gradually reached to the equilibrium state.

### 3.4. Adsorption Isotherm

SLACM was brought into contact with different concentrations of the solution (50–600 mg/L) in order to evaluate the effect of initial concentration on the adsorption capacity of SLACM for Cr(VI). Adsorption isotherms were used to evaluate the adsorption type and study the adsorption behavior under different equilibrium condition. The Langmuir model and Freundlich model were used to fit equilibrium experimental data of Cr(VI) adsorption on SLACM at temperatures of 20, 30 and 40 °C [37]. The isotherm models can be explained as follows.

This is an example of an equation:

(11) $$\mathrm{Langmuir}:\;q_{e}=\frac{K_{L}C_{e}q_{m}}{1+K_{L}C_{e}},$$

(12) $$R_{L}=\frac{1}{1+K_{L}C_{0}},$$

(13) $$\mathrm{Freundlich}:\;q_{e}=K_{F}C_{e}^{\frac{1}{n}},$$

where $q_{e}$ (mg/g) is the maximum adsorption capacity, $C_{e}$ (mg/L) is the equilibrium Cr(VI) concentration, $q_{m}$ (mg/g) is monolayer adsorption capacity, $K_{L}$ (L/mg) is the Langmuir affinity constant, $K_{F}$ (L/mg) is the Freundlich constant related to the adsorption capacity of the adsorbent and n is an empirical constant related to the adsorption intensity which varies with the heterogeneity of material.

**Figure 8:**
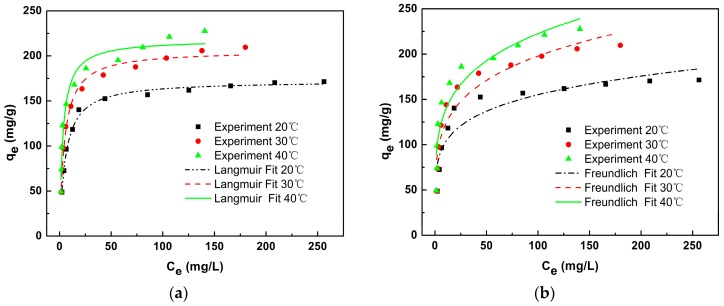
(**a**) Adsorption isotherm of Cr(VI) onto SLACM and Langmuir isotherm models fitting curves; (**b**) Adsorption isotherm of Cr(VI) onto SLACM and Freundlich isotherm models fitting curves.

The experimental data and non-linear fitting curves are shown in Figure 8. The fitting results and correlation coefficients are shown in Table 3. The Langmuir isotherm model is generally applicable to monolayer adsorption onto a homogeneous surface with a finite number of identical and equivalent sites. Meanwhile, it was supposed that the interaction forces between different adsorbates, between adsorbed molecules and adsorbent surface-active sites does not exist. The Freundlich isotherm model is generally applied to multilayer adsorption with interaction forces between different adsorbates, between adsorbed molecules and adsorbent surface-active sites [7]. As can be seen from the results in Table 3, Langmuir adsorption model has a higher correlation coefficient, and it can be better fitted to the adsorption type of SLACM for Cr(VI) than Freundlich model. Therefore, the isotherm model fitting results indicate that the adsorption process of SLACM for Cr(VI) was monolayer adsorption. The separation factor ($R_{L}$) is an important parameter for Langmuir isotherm model, which can be used to evaluate whether the adsorption process is favorable (0 < $R_{L}$ < 1), linear ($R_{L}$ = 1), or unfavorable ($R_{L}$ > 1) [37]. In this test Cr(VI) concentration range (50–600 mg/L), the $R_{L}$ values decreased from 0.1 to 0.0092 at 20 °C, from 0.0833 to 0.0075 at 30 °C, and from 0.0588 to 0.0052 at 40 °C. All of the $R_{L}$ values were greater than 0 but less than 1, and which were decreases with higher Cr(VI) initial concentration and higher adsorption temperature. The results indicate that the adsorption process of Cr(VI) onto SLACM was favorable in the concentration range studied, the higher Cr(VI) initial concentration and higher temperature was beneficial for adsorption. The equilibrium data fitted well with the Langmuir isotherm models, and the maximum adsorption capacity of Cr(VI) on SLACM was around 227.7 mg/g initial pH 2, 40 °C. The Cr(VI) adsorption capacities of the other adsorbents from biomass waste are listed in Table 4. within comparison with others, it can be seen that the SLACM had excellent adsorption capacity for the Cr(VI) removal from aqueous solution, and thus it could be a promising adsorbent.

## 4. Conclusions

Activated carbon microsphere was prepared from powdered SL without adding any kind of binder. The preparation process consisted of activated carbon precursor preparation by Mannich reaction and ZnCl_2_ activation. The results of the study indicated that the SLACM had excellent adsorption capacity for the removal of Cr(VI) from aqueous solution. It was found that the SL had a good Mannich reaction with aminated CMPS in creating ARM, and the ARM with good thermal stability was beneficial to the SLACM preparation. SLACM comprises tiny spherical particles with a well-developed porous structure. As a mesoporous material, the surface area and average pore size of spherical SLACM with a porous structure are 769.37 m^2^/g and 2.46 nm, respectively. The adsorption capacity of SLACM for Cr(VI) is greatly dependent on solution pH, and the acidity of the solution is beneficial to Cr(VI) adsorption. The adsorption kinetic results showed that the adsorption of SLACM for Cr(VI) mainly occurred during the initial stages of the adsorption process; the Elovich model was in better agreement with the experimental data to describe adsorption process than the PFO and PSO models. In the range of test temperatures, the adsorption capacity increased with increasing temperature. The equilibrium data fitted well with the Langmuir isotherm models, and the maximum adsorption capacity of Cr(VI) on SLACM was around 227.7 mg/g at an initial pH value of 2 and a temperature of 40 °C. Therefore, the SL could be considered to be a potential raw material for the production of activated carbon in removing the Cr(VI) from wastewater. The SLACM prepared in this work provides the possibility of wastewater treatment in the future.

## Figures and Tables

**Table 1:** Porous structure parameters of SLACM.

Sample	S_BET_ (m^2^/g)	S_mic_ (m^2^/g)	V_tot_ (cm^3^/g)	V_mic_ (cm^3^/g)	D_p_ (nm)
SLACM	769.37	639.28	0.47	0.26	2.46

**Table 2:** Adsorption kinetics model parameters of SLACM for Cr(VI).

Model	Parameters	Value
PFO	$q_{e}$	71.1
	$K_{L}$	106.75
	$R^{2}$	38.83
PSO	$q_{e}$	72.03
	$K_{2}$	3.59
	$R^{2}$	73.12
Elovich	α	5.81
	β	0.54
	$R^{2}$	95.63

**Table 3:** Adsorption isotherm parameters and correlation coefficients of SLACM for Cr(VI).

Temp °C	Langmuir			Freundlich		
	$q_{m}\;\mathrm{mg}/g$	$K_{L}\;L/\mathrm{mg}$	$R^{2}$	$K_{F}\;L/\mathrm{mg}$	1n L/mg	$R^{2}$
20	172.41	0.18	99.22	67.80	0.18	84.45
30	206.13	0.22	98.59	75.09	0.21	89.59
40	218.19	0.32	96.27	85.05	0.21	89.03

**Table 4:** The Cr(VI) adsorption capacities of the other adsorbents from biomass waste. Data from [33,34,39,44,45].

Adsorbent Precursor	Activating Agend	*q*_max_ (mg/g)	pH	Temp °C	Reference
Mixed waste tea	/	94.34	2.0	30	[44]
Coffee ground	/	87.72	2.0	30	[44]
Algal bloom	CO_2_	96	1.0	20	[39]
Glucose	KOH	332.53	2.0	25	[45]
Corn straw	KOH	175.44	3.0	25	[33]
Glucose monohydrate	H_2_O	117.2	2.0	30	[34]
SL	ZnCl_2_	227.7	2.0	40	This work
